# Quantifying superimposed protein flow dynamics in live cells using spatial filtering and spatiotemporal image correlation spectroscopy

**DOI:** 10.1111/jmi.13342

**Published:** 2024-07-04

**Authors:** Rodrigo A. Migueles‐Ramírez, Alessandra Cambi, Arnold Hayer, Paul W. Wiseman, Koen van den Dries

**Affiliations:** ^1^ Department of Quantitative Life Sciences McGill University Montreal Quebec Canada; ^2^ Department of Chemistry McGill University Montreal Quebec Canada; ^3^ Department of Physics McGill University Montreal Quebec Canada; ^4^ Department of Biology McGill University Montreal Quebec Canada; ^5^ Department of Medical BioSciences Radboud university medical center Nijmegen Netherlands

**Keywords:** actin, correlation spectroscopy, fluorescence microscopy, image analysis, myosin, podosomes, spatial filtering, STICS

## Abstract

Flow or collective movement is a frequently observed phenomenon for many cellular components including the cytoskeletal proteins actin and myosin. To study protein flow in living cells, we and others have previously used spatiotemporal image correlation spectroscopy (STICS) analysis on fluorescence microscopy image time series. Yet, in cells, multiple protein flows often occur simultaneously on different scales resulting in superimposed fluorescence intensity fluctuations that are challenging to separate using STICS. Here, we exploited the characteristic that distinct protein flows often occur at different spatial scales present in the image series to disentangle superimposed protein flow dynamics. We employed a newly developed and an established spatial filtering algorithm to alternatively accentuate or attenuate local image intensity heterogeneity across different spatial scales. Subsequently, we analysed the spatially filtered time series with STICS, allowing the quantification of two distinct superimposed flows within the image time series. As a proof of principle of our analysis approach, we used simulated fluorescence intensity fluctuations as well as time series of nonmuscle myosin II in endothelial cells and actin‐based podosomes in dendritic cells and revealed simultaneously occurring contiguous and noncontiguous flow dynamics in each of these systems. Altogether, this work extends the application of STICS for the quantification of multiple protein flow dynamics in complex biological systems including the actomyosin cytoskeleton.

## LAY DESCRIPTION

The collective movement of proteins, or protein flow, is a frequently observed phenomenon for many cellular components and drives key biological processes. For example, it is well established that the collective movement of cytoskeletal proteins such as actin at the cell front is crucial for controlling cell migration. To study protein flow in living cells, we and others have previously used spatiotemporal image correlation spectroscopy (STICS). STICS is a powerful microscopy technique that uses the correlation in pixel intensity fluctuations in recorded fluorescence image time series to calculate the velocity and directionality of protein flows. Such calculations are relatively straightforward when all the fluorescent proteins or protein complexes display flow at only one spatiotemporal scale. Yet, in living cells, proteins often display flow at multiple scales simultaneously, resulting in superimposed fluorescence intensity fluctuations that are challenging to separate using STICS alone. In this manuscript, we provide a potential solution for this challenge by exploiting the characteristic that distinct protein flows often occur at different spatial scales. We reasoned that processing the images with spatial image filters before the STICS analysis would allow to disentangle two superimposed flows. Specifically, we applied a Gaussian filter and developed a new Heterogeneity Map filter to either attenuate or accentuate local image fluorescence intensities across different spatial scales. Subsequently, we analysed the spatially filtered time series with STICS and demonstrated that the two superimposed flows in the image time series could now be distinguished. As a proof of principle of our analysis approach, we used simulated fluorescence intensity fluctuations as well as time series of nonmuscle myosin II in endothelial cells and actin‐based podosomes in dendritic cells and successfully untangled simultaneously occurring flow dynamics in each of these systems. Altogether, this work extends the application of STICS for the quantification of multiple protein flow dynamics in complex biological systems including the actomyosin cytoskeleton, among others.

## 1 INTRODUCTION

Live‐cell fluorescence microscopy provides key insights into the spatiotemporal organisation of proteins in living cells. A frequently observed phenomenon in fluorescence image time series is the coherent or collective movement of proteins, also called protein flow. Well‐known examples of protein flow include the retrograde movement of F‐actin at the leading edge of migrating cells^1^ and the centripetal movement of transmembrane receptors during immune synapse formation.^2^ Importantly, in fluorescence microscopy, protein flow leads to correlated fluorescence intensity fluctuations that can be quantitatively characterised by spatiotemporal image correlation spectroscopy (STICS).^3^ Specifically, STICS allows to spatially correlate fluorescence intensity values of a small region within an image with the corresponding region of an image acquired at a later point in time. By iteratively exploring all regions of the image and determining how the spatial correlation profiles evolve over time, flow vector fields can be generated and relevant parameters such as flow velocity magnitude and direction can be quantified.^3^

STICS has been applied to study the retrograde flow of F‐actin in T cells during immune synapse formation,^4^, ^5^, ^6^ the co‐transport of focal adhesion components in epithelial cells,^7^ and the mesoscale coordination of actin‐based podosomes in human dendritic cells.^8^, ^9^, ^10^ While these studies have provided valuable insights into the spatiotemporal dynamics of protein structure displacements in living cells, in many cases the fluorescence intensity fluctuations in image time series of biological systems are complex, as multiple protein flow components can coexist. For example, intensity fluctuations may be the result of the displacement of fluorescently labelled subcellular structures as well as the exchange of fluorescently labelled proteins between substructures. If both processes occur simultaneously in the same region of the cell, the image series will sample the superimposed flows, and these will be challenging to resolve individually via STICS as they will be averaged over in the ensemble spatial correlation inherent to the fluctuation analysis.

Disentangling superimposed protein flow dynamics in living systems is not trivial. If the protein flows occur over sufficiently distinct spatial regimes, a possible solution would be to acquire the image time series at different spatial resolutions. This, however, is a suboptimal approach since recording multiple time series is time‐consuming and acquisition parameters such as the objective lens may need to be changed in between recordings. Also, it is increasingly common to use super‐resolution for live‐cell imaging, including structured illumination microscopy^12^ and Airyscan imaging,^13^ which permits the direct correlation of the nanoscale organisation of proteins with the STICS flow patterns.^10^ Most importantly, recording multiple time series does not solve the conundrum of simultaneously characterising multiple dynamic processes spanning different scales in the same cell.

Using STICS, it is possible to increase or decrease spatial sampling of fluorescence fluctuations from which a field is calculated by modulating the size of the region of interest (ROI), but the fluorescence fluctuations are still measured with the same spatial resolution. Similarly, the time of interest (TOI) can be adjusted to encompass eventful periods or to minimise the contributions of immobile components within the image series. Yet, when the spatial frequencies that characterise different superimposed flow dynamics are on significantly different scales, these spatial or temporal sampling adjustments are insufficient to distinguish them as they will still manifest in the images.^10^ Furthermore, depending on the relative brightness of the imaged superimposed flows, one can dominate the STICS results due to a quadratic brightness weighting in the correlation function, obscuring the contribution of the less bright population. It would therefore be advantageous to develop image processing procedures to simultaneously measure superimposed flows on different spatial scales from fluorescence image time series.

Here, we provide a strategy to quantify superimposed flow dynamics in fluorescence image time series using spatial filtering prior to the STICS analysis. Specifically, we employ a normalising kernel and a 2D Gaussian function to highlight or reduce local heterogeneity of the image fluorescence intensity values at variable spatial scales. As a proof of principle, we used our strategy to extract multiple protein flow dynamics from image time series of nonmuscle myosin II (NMII) in endothelial cells and actin‐based podosomes in dendritic cells. Altogether, our work broadens the potential applications of STICS to study the spatiotemporal organisation of many complex biological systems including cytoskeletal rearrangements and intracellular signalling.

## 2 RESULTS

### 2.1 Contiguous and noncontiguous fluorescence intensity fluctuations

**FIGURE 1:**
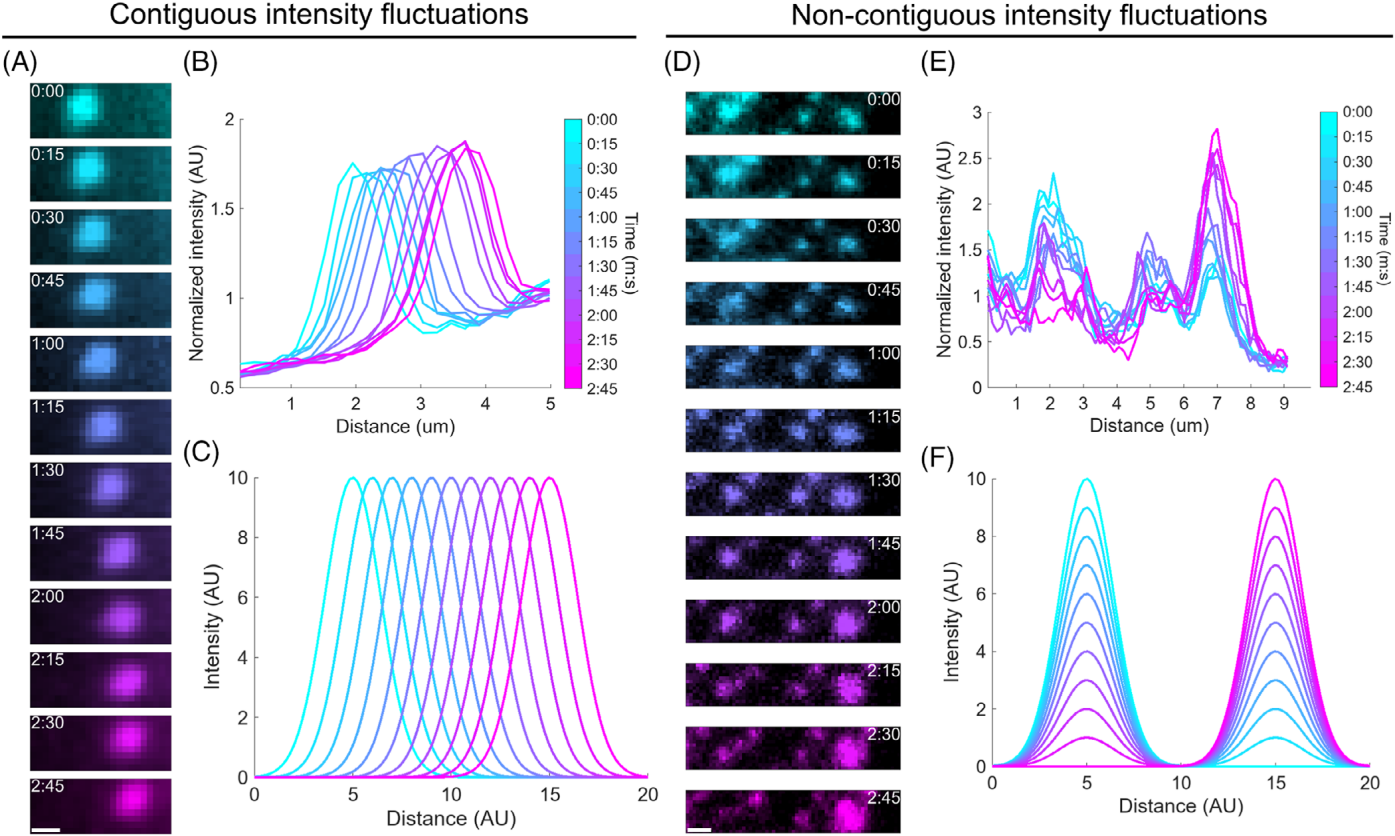
Experimental examples and schematic representations of contiguous and noncontiguous fluorescence intensity fluctuations. (A) Image time series of a vesicle containing mCherry‐MYL9 experiencing displacement. Scale bar, 1 µm. (B) Fluorescence intensity profiles of each frame in A. Each line reports the average intensity of an image in A along the *y*‐axis as a function of the distance along the *x*‐axis. (C) Schematic representation of the evolution of Gaussian fluorescence intensity profiles simulating a displacement like the one observed in A and B. (D) Image time series of podosomes labelled with LifeAct‐GFP undergoing F‐actin content fluctuations over time. Scale bar, 1 µm. (E) Fluorescence intensity profiles of each frame in D determined as in B. (F) Schematic representation of the evolution of Gaussian fluorescence intensity profiles simulating a propagation like the one observed in D and E.

In this work, we consider two types of fluorescence intensity fluctuations that are apparent in fluorescence image time series of living cells. First, there are spatially continuous or contiguous fluorescence intensity fluctuations which can for example result from the directional displacement of fluorescently labelled structures across space (Figure 1A–C). When a fluorescently labelled vesicle moves across the cell, the corresponding pixels record the sequential intensity fluctuations due to the flow transport with respect to the neighbouring pixels (Figure 1B). The location of the structure changes, but it mostly preserves the same intensity profile as it moves (Figure 1C). Second, there are spatially noncontinuous or noncontiguous fluorescence intensity fluctuations, which can, for instance, result from the accumulation or dissociation of fluorescently labelled molecules as they bind or unbind to different subcellular structures (Figure 1D–F). When fluorescently labelled molecules dissociate from one structure and subsequently associate to a neighbouring structure, the corresponding pixels show an intensity decrease in one area accompanied by an intensity increase in the adjacent area (Figure 1E). The location of the structures does not change much, but their composition in terms of fluorescently labelled molecules fluctuates in a spatiotemporally correlated manner (Figure 1F).

### 2.2 Increasing or reducing local fluorescence heterogeneity using spatial filtering

STICS can extract flow dynamics from both contiguous and noncontiguous fluorescence intensity fluctuations.^8^ When superimposed in one image time series, noncontiguous fluorescence intensity fluctuations usually have larger spatial scales as compared to contiguous fluctuations since the contiguous displacement of structures occurs on shorter distance scales as compared to the exchange of proteins between neighbouring structures, which can manifest as noncontiguous intensity fluctuations. We therefore reasoned that distinct image spatial filtering algorithms could be applied to separate overlapping contiguous and noncontiguous fluorescence intensity fluctuations.

**FIGURE 2:**
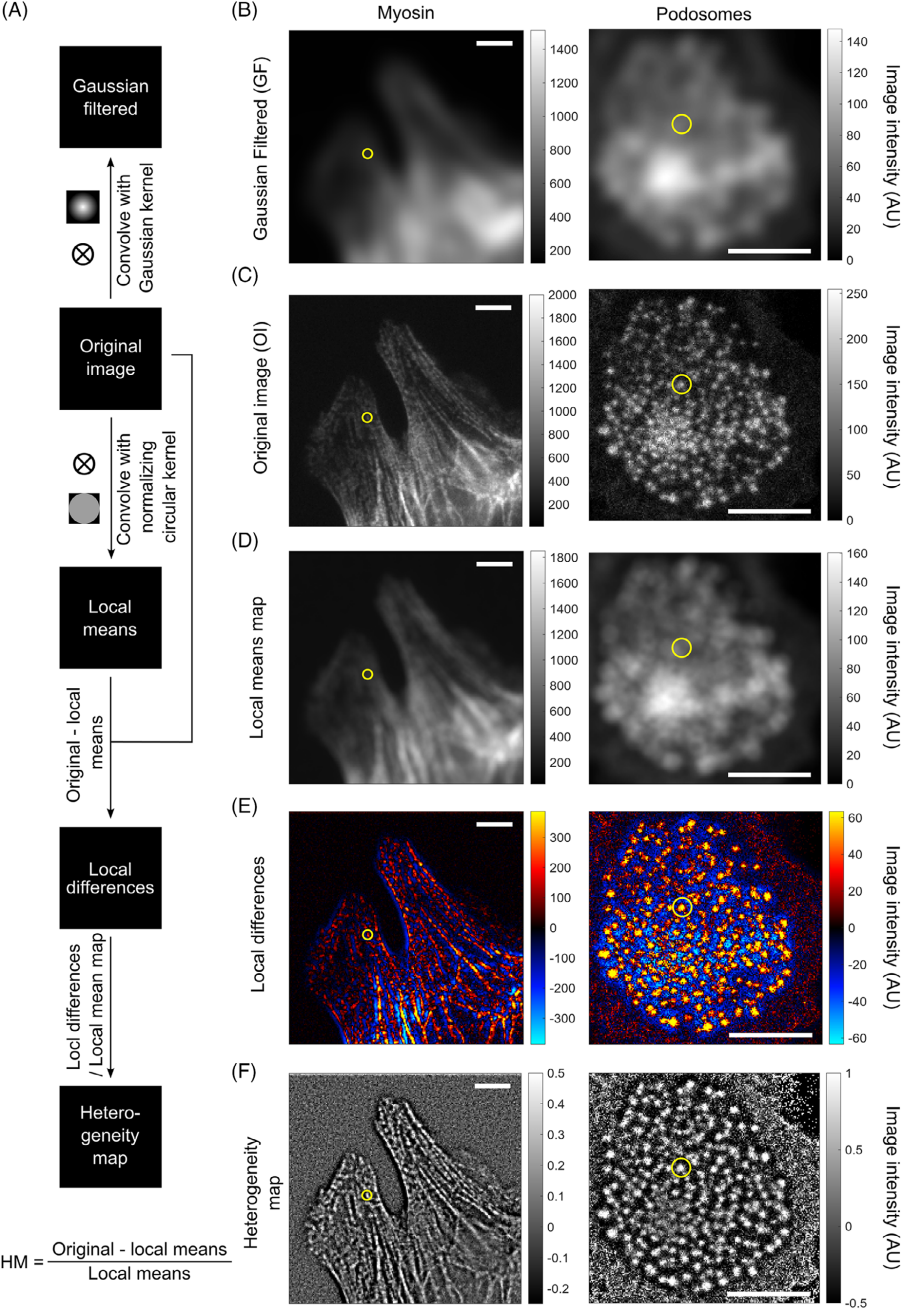
Spatial filtering to decrease or increase local heterogeneity. (A) Schematic overview of the mathematical operations applied to the original image to retrieve the Gaussian filtered image as well as the heterogeneity map image. (B–F) Representative unfiltered and filtered images of HT‐HUVEC stably expressing mRuby3‐MYL9 (myosin regulatory light chain, a reporter for NMII) (left column), or dendritic cells transiently expressing Lifeact‐GFP to visualise F‐actin‐based podosomes (right column). For the heterogeneity map, also the local means and local differences are shown as intermediates. For the Gaussian filtered images, a standard deviation of σ = 7 pixels (∼13.7 µm in diameter) was used and for the heterogeneity map a kernel size of 8 pixels (∼1.7 µm) was used for myosin and 16 pixels (∼3.4 µm) for podosomes. Scale bars: 10 µm.

To detect noncontiguous intensity fluctuations, we applied a Gaussian spatial filter (GF) on the original image (OI) to virtually increase the size of the point spread function (PSF) computationally (Figure 2A–C). This served to correlate fluorescence intensities between adjacent structures over which the flow is sampled. Doing so allowed us to interpolate the intensity between spatially separated structures, preserving the differences in fluorescence intensity amplitudes across regions, and minimised the contribution of image features reflecting the sizes and shapes of the subcellular structures in the image (Figure 2A–C, Video S1).

In contrast, if the contiguous fluorescence intensity fluctuations caused by the displacement of subcellular structures are the feature of interest, then the contributions of image features that reveal their locations and shapes should be preserved while we suppress the large‐scale spatial differences in fluorescence intensity. To do this, we developed a new algorithm, which preserves the local spatial heterogeneity that defines the texture of the image, regardless of the absolute intensity and thus the global differences in fluorescence intensity (Figure 2C–F). By normalising the intensity of each pixel to the average intensity of pixels in its neighbourhood, pixels with significantly higher intensity values with respect to their local neighbourhood have greater heterogeneity values (and vice versa). As such, the filtered image is a heterogeneity map (HM) of regions with significantly greater pixel intensities relative to local average intensity values (Figure 2F).

Taken together, we expect that reducing the local fluorescence heterogeneity in space enables the quantification of noncontiguous fluorescence intensity fluctuations, whereas increasing it can remove the contribution of noncontiguous fluctuations and therefore improve the quantification of contiguous fluorescence intensity fluctuations. To test our spatial filtering approach, we first evaluated its effectiveness on videos with simulated intensity fluctuations and next, chose two biological systems in which both contiguous and noncontiguous flow dynamics coexist, with distinct relative contributions, and sought to separately quantify them.

### 2.3 Detecting superimposed flows in simulated fluorescence intensity fluctuations

**FIGURE 3:**
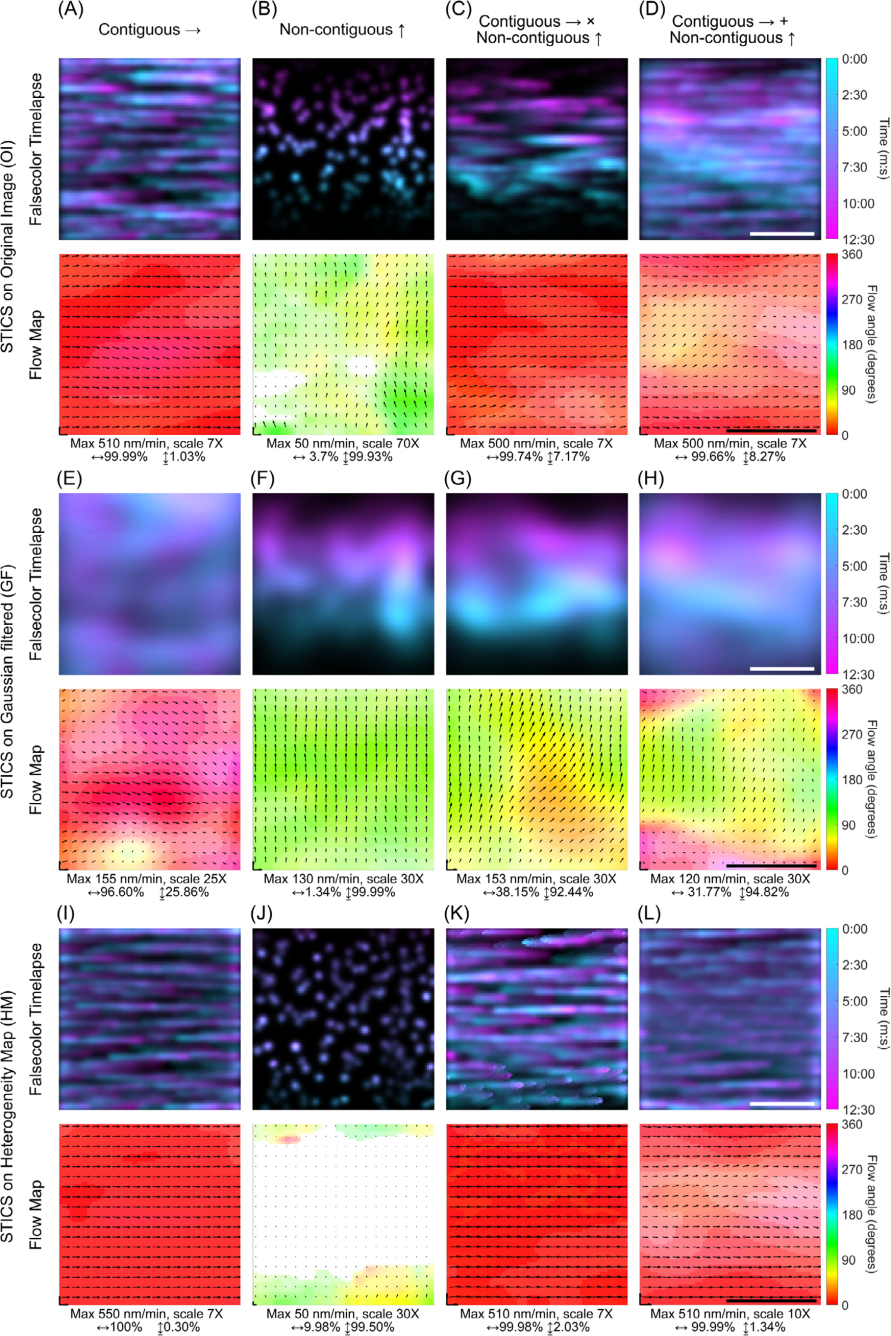
Detecting contiguous and noncontiguous fluorescence intensity fluctuations in simulated fluorescence image series. (A–D) Temporal colour‐coded image series of simulated intensity fluctuations that are contiguous (A), noncontiguous (B), contiguous multiplied by noncontiguous (C), and contiguous added to noncontiguous. Bottom row panels show the STICS average vector fields across frames obtained from the respective image series. (E–H) Temporal colour‐coded Gaussian filtered image series of the simulations shown in A–D. Bottom row panels show the STICS average vector fields across frames obtained from the respective Gaussian filtered image series. (I–L) Temporal colour‐coded heterogeneity map filtered image series of the simulations shown in A–D. Bottom row panels show the STICS average vector fields across frames obtained from the respective Heterogeneity map image series. Underneath each vector field the maximum vector velocity is shown, the scaling factor used for visualising the vectors as well as a percentage the median cosine between the angle of each vector and the horizontal (0°) or vertical (90°) vectors, where 100% indicates perfect alignment and 0% no alignment.

To first test our filtering approach in a system that allows precise control of the direction and speed of the superimposed flows, we generated videos with simulated contiguous and noncontiguous fluorescence intensity fluctuations or a mix of both (Video S2). To properly mimic typical structures observed in living cells, we generated fluorescent particles with a radius of ∼0.5 µm, a diffusive wave with a radius of ∼2 µm, and flow speeds of ∼0.5 µm/min. Furthermore, to make the difference between the contiguous and noncontiguous flows as clear as possible, the contiguous flow travels horizontally through the image series (Figure 3A), while the contiguous travels vertically (Figure 3B). To generate simulated image series with a mix of both types of flows, we first performed a multiplication of the two signals (Figure 3C), mimicking cellular structures that simultaneously move and change intensity over time as we have for example observed before for actin‐based podosomes in dendritic cells.^8^ Lastly, we generated simulated image series that contained an addition of a diffusive vertical flow with particles presenting horizontal contiguous flow, mimicking a dense molecular flow traveling through the cell and exchanging molecules with multimolecular complexes (Figure 3D).

To test the general ability of STICS to detect these different types of simulated flows, we first analysed the unfiltered videos. As expected, STICS was able to detect both the noncontiguous and contiguous fluorescence intensity fluctuations when only one type was present in the simulations, as indicated by the horizontal and vertical vector directions, respectively (Figure 3A and B). Yet, when STICS was applied to a simulated image series with a mix of intensity fluctuations, STICS typically detected only the most dominant wave type or, in some regions, generated a mix of vectors for which the directionality was determined by both types of fluctuations simultaneously (Figure 3C and D).

To assess how the filtering affected the STICS output, we first filtered the image series of the individual waves. As expected, the HM did not significantly influence the detection of the contiguous wave while the GF left the detection of the noncontiguous wave mostly unaffected (Figure 3F and I). In contrast, when the GF was applied to the contiguous wave and the HM was applied to the noncontiguous wave, the detection of the waves was strongly impaired (Figure 3E and J), explained by the fact that the fluctuations were mostly absent due to the spatial filtering. Lastly, we applied the GF and the HM to the videos that contained a mix of contiguous and noncontiguous intensity fluctuations (Figure 3G, H, K, and L). From this, we concluded that indeed, the different types of fluorescence intensity fluctuations were separately detected using our approach. For the GF movies, the vectors almost exclusively pointed in the direction of the noncontiguous flow (Figure 3G and H), which was barely detected in the STICS analysis of the OI. By contrast, for the heterogeneity map videos, the vectors all pointed in the direction of the contiguous flow (Figure 3K and L). Altogether, these results indicate that our filtering approach is able to distinctly detect superimposed fluorescence intensity fluctuations in simulated data that contain a mix of contiguous and noncontiguous fluctuations, as frequently observed in living cells.

### 2.4 Nonmuscle myosin II contiguous flow dynamics along actomyosin fibres

**FIGURE 4:**
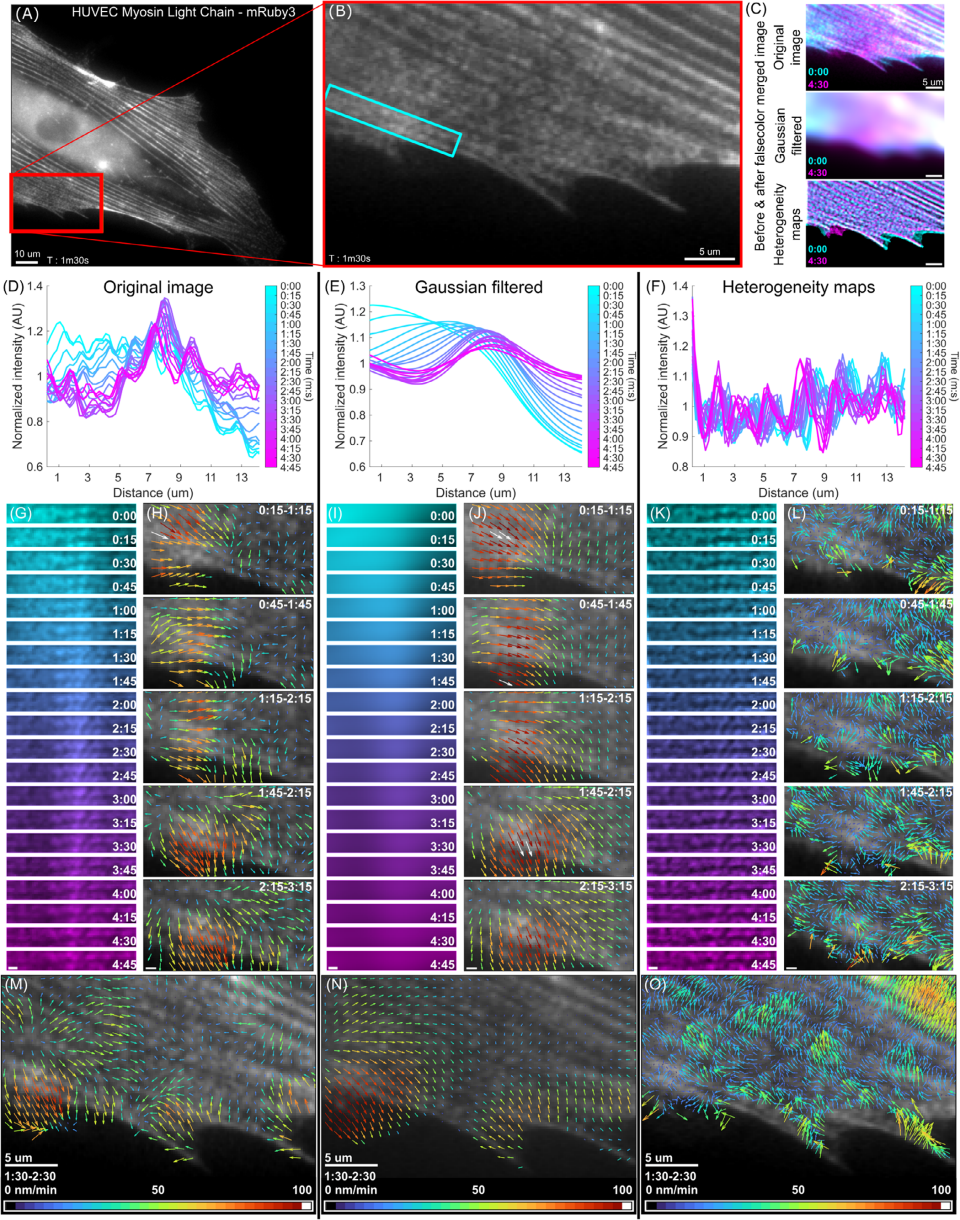
Quantifying contiguous and noncontiguous flow dynamics of NMII using STICS. (A) HT‐HUVEC stably expressing mRuby3‐MYL9 featuring NMII puncta along actomyosin bundles. Scale bar, 10 µm. (B) Magnification of the highlighted region in A showing a wave of fluorescence intensity fluctuations traveling along actomyosin bundles. (C) False‐colour merged images showing regions with increased intensity at the beginning (cyan) and at the end (magenta) of the sequence in the original, Gaussian filtered, and heterogeneity map images. Scale bars 5 µm. (D–F) Rectangular kymographs showing the progression of the intensity wave from left to right as well as the modest movement of myosin puncta from right to left. Kymographs were obtained by averaging along the columns of each montage image. (G, I, K) Montage series for D, E, and F, respectively. Scale bars 1 µm. (H, J, M, N) Original NMII images overlaid with vector fields obtained by applying STICS to either the original (H, M) or the Gaussian‐filtered (J, N) image series. ROI size 32 pixels, TOI size 5 frames (1 m 15 s). Scale bars 1 µm (H, J) and 5 µm (M, N). (L, O) Original myosin images overlaid with the vector fields obtained by applying STICS to the heterogeneity map image series. ROI size 16 pixels, TOI size 5 frames (1 m 15 s). Scale bars 1 µm (L) and 5 µm (O).

As a first biological system, we investigated the spatiotemporal organisation of nonmuscle myosin II (NMII) in hTERT‐immortalised human umbilical vein endothelial cells (HT‐HUVECs). NMII is a large hexameric protein complex consisting of two heavy chains and four light chains.^11^ Upon phosphorylation of the regulatory light chains, NMII changes conformation from a closed to an open state, enabling the assembly of the hexamers into bipolar filaments and binding to F‐actin.^11^ To visualise NMII, we used HT‐HUVECs stably expressing myosin regulatory light chain (MYL9) fused to mRuby3 (mRuby3‐MYL9), which reports both inactive and active conformations of NMII. In epifluorescence microscopy, the active NMII bipolar filaments associated with F‐actin appear as diffraction‐limited puncta, whereas inactive NMII is diffusely distributed throughout the cytoplasm (Figure 4A). Importantly, in image time series, NMII puncta undergo lateral displacements along actomyosin bundles as they contract, while simultaneously presenting sequential changes in intensity at different regions along the actomyosin bundles, which are presumably due to the binding of diffuse NMII hexamers to existing NMII bipolar filaments^12^, ^13^ (Figure 4B, Video S3). Together, these two processes resulted in contiguous flow of NMII puncta at the microscale (∼1–2 µm) as well as the noncontiguous flow dynamics of NMII puncta intensity fluctuations at the mesoscale (∼5–10 µm), each representing a layer of flow we aimed to separate.

To distinguish these two flow dynamics, we first identified a region visibly featuring both contiguous and noncontiguous flow dynamics (Video S3). We then applied our spatial filtering approach and compared the calculated STICS results using either the OI, the GF, or the HM time series. False‐colour merged images before and after the visible passage of noncontiguous flow indicated that most of the intensity changes observed in the OI were retained in the GF images, while the HM retained the local spatial patterns reporting puncta locations (Figure 4C). To investigate this further, we isolated a region of interest (Figure 4B) and created montages with either the OI, GF or HM time series (Figure 4G, I, and K). The region of interest visibly displayed propagation of puncta intensity in one direction and a slow displacement of NMII puncta in the opposite direction (Video S4). Rectangular kymographs of the selected region showed that, while the OI intensity profiles harboured a mix of patterns and intensity fluctuations, the GF profiles contained large scale intensity fluctuations, whereas the local HM profiles predominantly highlighted the displacement of local intensity profiles (Figure 4D, E, and F).

We next tested whether our filtering approach enabled the separate quantification of both flow dynamics using STICS. In the STICS vector fields from the OI, the intensity fluctuations dominated the STICS correlation functions, mostly reflecting the noncontiguous flow dynamics and masking the myosin puncta displacements (Figure 4H and M, Video S5). Reducing the ROI size did not improve the results significantly, as it resulted in an ambiguous mix of vectors variously reporting contiguous and noncontiguous flow (Video S6). In comparison, STICS vectors generated using the GF image series all pointed in the direction of the noncontiguous flow, mostly revealing propagating waves of myosin intensity (Figure 4J and N, Video S7). Furthermore, applying STICS to the local HM resulted in vectors that pointed in the opposite direction of the noncontiguous flow, in agreement with the contiguous flow of puncta observed in the time series (Figure 4L and O, Video S8). To note, we adjusted the ROI size to the characteristic puncta size, which improved the resolution of STICS vector fields as compared to a larger ROI size (compare Videos S8 and S9) and did not affect the overall direction of the detected contiguous flow.

Together, these observations demonstrate that using the spatial filtering algorithms in combination with appropriate STICS parameter selection enables the separate detection of both layers of flow dynamics. This approach permitted the quantification of contiguous NMII puncta flow, which was otherwise precluded by the presence of the noncontiguous NMII flow.

### 2.5 Podosome lateral movement and F‐actin intensity fluctuations

**FIGURE 5:**
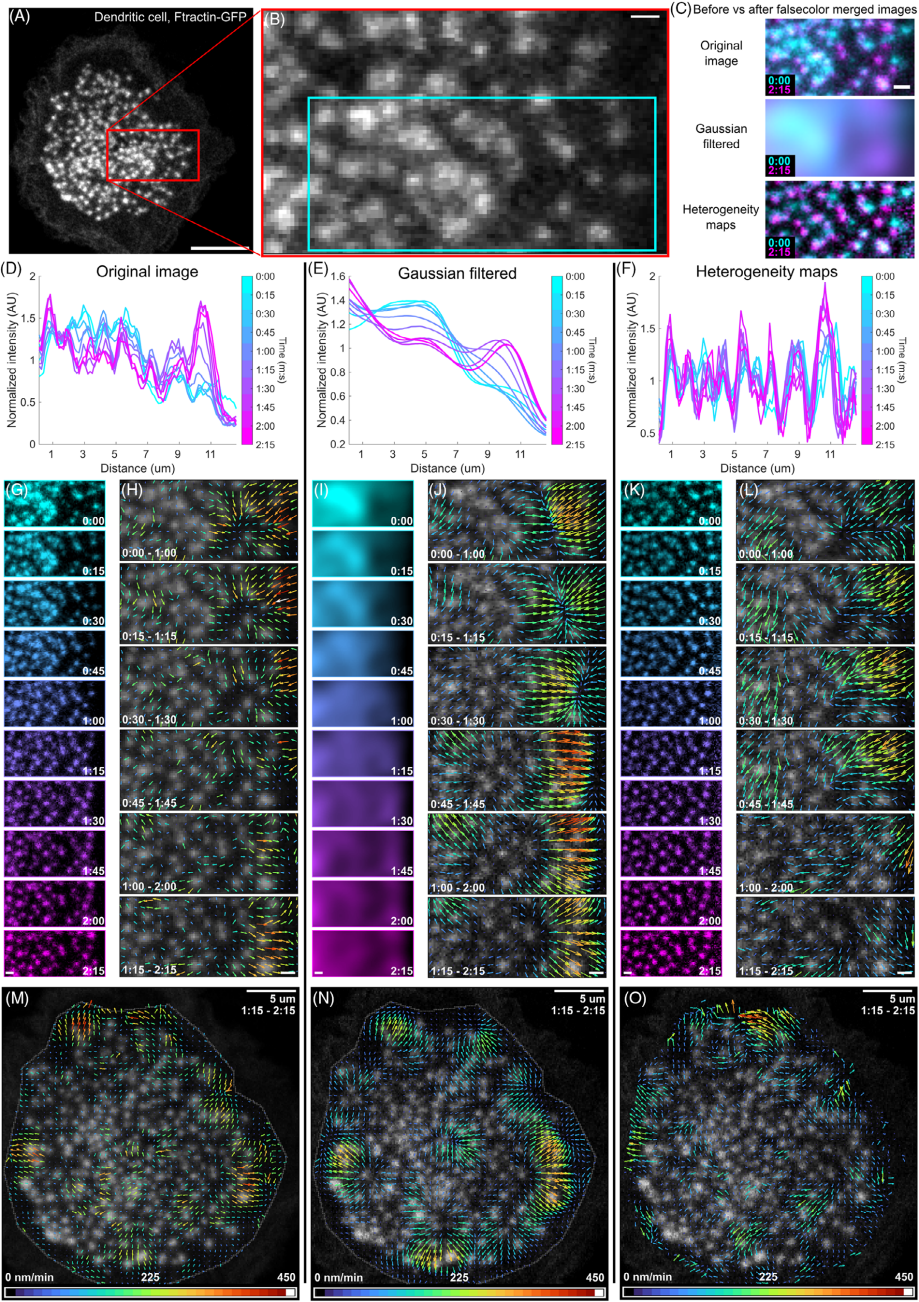
Quantifying podosome contiguous displacement and noncontiguous F‐actin intensity propagation flows in dendritic cells using STICS. (A) Dendritic cell transiently expressing LifeAct‐GFP featuring podosomes. Scale bar 10 µm. (B) Magnified view of A showing a podosome cluster. Scale bar, 1 µm. (C) False colour merged images showing regions with increased intensity at the beginning (cyan) and at the end (magenta) of the sequence in the original, Gaussian filtered, and heterogeneity map images. Scale bars, 1 µm. (D–F) Rectangular kymographs showing the progression of the intensity wave from left to right as well as the modest movement of some podosomes from right to left. Profiles were obtained by averaging along the columns of each image in the montage series. (G, I, K) Montage series for D, E, and F, respectively. Scale bars 1 µm. Scale bars, 1 µm. (H, J, L, M–O) Original images overlaid with the vector fields obtained by applying STICS to either the original (H, M) the Gaussian‐filtered (J, N), or the heterogeneity map image series (L, O). ROI size 32 pixels (4.48 µm), TOI size 5 frames (1 m 15 s). Scale bars, 1 µm (H, J, L) and 5 µm (M, N, O).

As a second biological system to test our approach, we examined the spatiotemporal organisation of podosomes in human DCs (Figure 5A, Video S10). Podosomes are micron‐sized actin‐rich structures present at the adhesion surface of DCs and macrophages, among others, and are involved in cell migration across tissue barriers.^14^ They play a role in substrate stiffness sensing though cycles of actin polymerisation and depolymerisation against the plasma membrane.^15^ As their structure is remodelled, podosomes undergo modest lateral displacements in the range of 1–3 µm over the course of several minutes as well as continuous F‐actin content fluctuations with a typical period of 1–3 min.^15^ These F‐actin fluctuations are correlated between neighbouring podosomes due to propagating actin flows throughout the cluster that extend up to 10–20 µm.^8^ To visualise podosomes, we transiently transfected DCs with the filamentous actin marker LifeAct‐GFP and acquired image time series using confocal microscopy. The observed changes in fluorescence intensity reflect both the contiguous lateral displacement flow of individual podosomes and the noncontiguous flow caused by sequential assembly and disassembly of actin in neighbouring podosome structures.

To separate the two layers of flow dynamics, we isolated regions presenting visually detectable noncontiguous flow waves (Figure 5B, Video S11). We applied our filtering algorithms (Video S12) and compared the images at the beginning with those at the end of a noncontiguous actin wave by merging them (Figure 5C). As with the NMII case, the OI presented both contiguous and noncontiguous fluorescence intensity changes reflecting the lateral movement and F‐actin content fluctuations of podosomes. In comparison, the GF images captured most of the podosome intensity fluctuations that extend throughout the cluster, while the local HM images reported only the micron scale lateral displacements of the podosomes. These observations were then visualised using rectangular kymographs (Figure 5D–F) of montages with the OI, GF and HM images, respectively. This showed that the overall F‐actin profiles observed in the OI time series (Figure 5G) contained contributions from both the intensity values found in the GF profiles (Figure 5I) and the podosome location patterns found in the HM images (Figure 5K).

We next evaluated the effect our filtering algorithms had on the capacity of STICS vector fields to capture each layer separately. Applying STICS to the OI time series resulted in regions with high differences in angle and magnitude among neighbouring vectors, overall capturing a mixture of podosome displacements and fluorescence intensity fluctuations (Figure 5H and M, Video S13). Comparing these with STICS vectors from the GF time series (Figure 5J and N) suggested that most vectors calculated from OI time series were representing overall intensity fluctuations. Modulating the ROI size did not significantly improve our ability to distinguish the contribution of each flow layer to the STICS results (Video S14). STICS vector fields from GF time series were aligned with the visible actin wave propagation from left to right among neighbouring podosomes in the cluster (Figure 5J, Videos 15 and 16). Interestingly, in the HM time series, STICS detected vectors pointing in the opposite direction in the same region of interest, reflecting the lateral displacement of podosomes (Figure 5L and O, Video S17). Therefore, applying spatial filtering before STICS allowed the detection and quantification of distinct flow patterns involved in collective podosome dynamics.

## 3 DISCUSSION

We and others have previously demonstrated the application of STICS to measure the spatiotemporal dynamics of various cell biological processes in living cells.^4^, ^5^, ^8^, ^9^, ^15^ In these studies, the correlation analysis was implemented on image series after immobile fraction subtraction. However, the complex cellular dynamics often contain multiple flow components in distinct spatiotemporal regimes which are challenging to quantify separately. In this technical note, we present a spatial filtering‐based strategy that extends the applicability of STICS to detect multiple flow patterns in a single fluorescence microscopy image time series. By tuning the level of spatial heterogeneity in the filtered image time series, we show that it is possible to disentangle contiguous from noncontiguous flow patterns in simulated image series containing multiple types of fluorescence intensity fluctuations as well as two different biological systems, NMII dynamics in endothelial cells and actin‐based podosomes in dendritic cells, thereby broadening the potential of STICS to study complex dynamic processes in cells.

In this work, we considered the existence of contiguous and noncontiguous fluorescence intensity fluctuations in time‐lapse videos of living cells. For the interpretation of the STICS output parameters, it is, however, important to note that the apparent noncontiguous fluctuations observed in the image time series could be the result of an underlying contiguous molecular process that is not detected by the microscope due to the temporal sampling rate or low fluorescence intensity. A correlated increase and decrease in fluorescence intensity of adjacent structures could be the result of fluorescently labelled particles unbinding from one structure, diffusing between them and binding to the other.^10^ This molecular displacement is, however, not detected using conventional imaging and it therefore only appears as noncontiguous, temporally correlated fluorescence intensity fluctuations of adjacent structures. Similarly, contiguous intensity fluctuations may be caused by noncontiguous molecular processes. For example, in structures that undergo depolymerisation/polymerisation dynamics including actin‐based structures, enhanced polymerisation at one side of the structure and decreased polymerisation at the other would be a noncontiguous molecular process that leads to apparent contiguous fluorescence intensity fluctuations. For interpreting the STICS results and derive molecular conclusions, we emphasise that it is important to include the ensemble of information available about the proteins of interest.

Importantly, while the cells analysed in the current manuscript were nonmigratory within the timeframe of imaging, potential cell movements may further increase the complexity of the fluorescence intensity fluctuations in the time‐lapse videos depending on the cell speed and directionality. To properly interpret the intracellular dynamics with the cell as the frame of reference, we therefore suggest to first register the acquired video to correct for cell movement and subsequently perform the spatial filtering and STICS analysis.

To highlight contiguous flow patterns, we developed heterogeneity maps (HM), a new spatial filtering algorithm that calculates the normalised pixel intensity with respect to its local neighbourhood. Its application results in an image composed of patterns in which global differences in intensity are almost completely lost, while local differences are preserved and accentuated. To our knowledge, this type of filtering algorithm has not been used in such context before. Unlike sharpening (unsharp mask filtering) approaches, in which a blurred version is subtracted from the original image,^16^ our heterogeneity mapping algorithm normalises the fluorescence intensity with the mean local intensity, highlighting fluorescent aggregates, even in dim regions of the image. In contrast with other first or second order differential filters, our approach does not explicitly consider the intensity between adjacent pixels, but rather the relative value of the central pixel with its neighbourhood locally in a circular region.^16^ In addition to highlighting NMII foci and podosomes as shown in this manuscript, heterogeneity mapping could be applied to accentuate other biological structures in fluorescence microscopy images such as cell–cell junctions or organelles.

To optimise the application of the heterogeneity map or the Gaussian filtering for time‐lapse videos of other biological samples than those described in this manuscript, the kernel size (for the heterogeneity map) or sigma (for the Gaussian filtering) must be adjusted to the spatial characteristics of the structures of interest. For the heterogeneity map, the kernel size ideally encompasses a single structure of interest and as much space between adjacent structures as possible. Choosing a kernel size that is too small may highlight structures that are smaller than those of interest and choosing a kernel size that is too high will not sufficiently emphasise the local heterogeneity. For the Gaussian filtering, the sigma is ideally larger than the distance between adjacent structures. Choosing a sigma that is too small will insufficiently filter out the contiguous fluctuations and choosing a sigma that is too high will average out all fluorescence intensity fluctuations impairing the detection of the noncontiguous flow. For all practical purposes, we advise users to probe a small range of kernel or sigma values and assess the STICS performance qualitatively and quantitatively.

The spatiotemporal regimes that are analysed with STICS can be modulated by selecting a specific region of interest (ROI) size and time of interest (TOI) for the correlation function analysis.^8^ A relatively large ROI size samples features across a larger area within the image time series, whereas a small ROI size samples features over a smaller local area. Similarly, a relatively large TOI size samples slow events, whereas a small TOI size captures fast, rapidly changing events.^8^ Importantly, the selection of an appropriate ROI and TOI size significantly influences the outcome of the STICS analysis as the correlation function uncertainty depends on the square root of the number of independent fluctuations sampled.^17^ A suboptimal ROI or TOI size may therefore result in fewer or erroneous vectors derived from the STICS correlation functions due to insufficient sampling of fluorescence fluctuations. For instance, to obtain the results presented here, we decreased the STICS analysis ROI size of the NMII heterogeneity maps as compared to the Gaussian filtered images to align with the small‐scale lateral displacements of the NMII foci. For future applications, it is therefore essential to optimise the ROI and TOI selection in addition to the spatial filtering kernel to properly extract the relevant protein flow parameters.

In conclusion, we are introducing a new image filtering approach that enables STICS to probe dynamics on different spatiotemporal regimes in fluorescence image time series. Our approach opens the door for STICS to quantify complex biological systems that are characterised by multiple protein flow patterns such as the reorganisation of the cytoskeleton, the plasma membrane or intracellular signalling pathways.

## 4 MATERIAL AND METHODS

### 4.1 Cell culture

The hTERT‐immortalised human umbilical vein endothelial cells (HT‐HUVECs) used were generated by stable transduction of primary HUVEC from mixed (male, female) donors with hTERT.^18^ Cells were cultured in EndoGRO VEGF (Millipore Sigma, SCME002), supplemented with Hygromycin (50 µg/mL). HT‐HUVEC stably expressing the reporter constructs mCherry‐MYL9 (Figure 1A and B) or mRuby3‐MYL9 (Figures 2 and 3) were generated by lentiviral transduction of HT‐HUVEC with pLV‐MYL9‐mCherry‐IRES‐NeoR or pLV‐MYL9‐mRuby3‐IRES‐Neo,^19^ followed by antibiotic selection (0.5 mg/mL G418). Stably transduced cells were maintained in the presence of G418.

### 4.2 Simulations of fluorescence intensity fluctuations

Simulations were generated using custom MATLAB code. Each particle was simulated as a particle with a point spread function (PSF) with a 1/*e*2 radius of 500 nm for an apparent diameter of almost 1 µm. Particle locations were randomly sampled from a uniform distribution. Particle density was ∼5 particles per µm^2^, resulting in 200 particles in a 100 × 100 px canvas with a pixel size of 0.14 µm. Each particle was simulated to emit 400 photons per second (integral of the PSF) and all particles had the same intensity, without photo bleaching or blinking. Apparently, bright particles are due to two or more particles close to each other. Particle locations were then subjected to either no displacement or to a contiguous displacement towards the right at a constant speed of 500 nm/min. To simulate the noncontiguous fluorescence fluctuations, a second layer was simulated. In this layer, 100 particles with a PSF radius of 2 µm were randomly distributed along a horizontal line located 1/3 of the canvas from bottom to top. The image series were then generated by either adding or multiplying the contiguously moving layer with the noncontiguous layer. For clarity and simplicity, no noise was included in the simulations.

### 4.3 Live‐cell imaging of DCs

Day 6 DCs were generated from peripheral blood mononuclear cells as previously described.^20^ The day before imaging, 1 × 10^6^ DCs were transiently transfected with 6 µg plasmid DNA coding for LifeAct‐GFP using the Neon Transfection System (Life Technologies). Cells were subsequently cultured overnight in WillCo dishes (WillCo Wells B.V.) and directly before imaging, the medium was replaced by culture medium without phenol red to avoid background fluorescence. Image time series were acquired on a Leica SP8 (pixel size 0.14 µm, frame interval 15 s), equipped with a HC PL APO CS2 63×/1.2 NA water immersion objective, and using HyD detectors operated in photon counting mode with time gating (0.3−6 ns). GFP was excited by 488 nm light and emission light was filtered by the AOBS set at 500−550 nm.

### 4.4 Live‐cell imaging of HUVECs

HUVECs were imaged as previously described.^19^ Briefly, HUVECs were seeded on 96‐well optical grade glass‐bottom plates (Cellvis P96‐1.5H‐N) previously coated with type I bovine collagen (31 µg/mL in PBS for 2–4 h at 37°C). For live‐cell imaging, cells were incubated using a CO_2_‐independent live‐cell imaging solution (LIS) with low autofluorescence (125 mM NaCl, 5 mM KCl, 1.5 mM MgCl_2_, 1.5 mM CaCl_2_, 10 mM D‐glucose, 20 mM HEPES pH 7.4, 1% FBS and 5 ng/mL bFGF). To avoid evaporation, plates were sealed with aluminium microplate seals (PolarSeal, Thomas Scientific 1152A34). A fully automated widefield fluorescence microscope system (Nikon TI2‐E), controlled using Nikon NIS Elements AR software, was used for live cell imaging. We employed a 60× 1.4 NA Plan Apo oil immersion objective (Nikon). Excitation was done using a multispectral LED light source (SpectraX, Lumencor). Detection was performed using a custom‐integrated high‐speed emission filter wheel (HS‐1025, FLI), and a sCMOS camera (Orca Fusion‐BT, Hamamatsu). Images were acquired using a pixel size of 0.2146 µm, and a frame interval of 15 s.

### 4.5 Image analysis

Image analysis was performed using ImageJ Fiji and MATLAB 2023b. Images were first registered using a Fourier‐based homemade image registration algorithm in MATLAB. Resulting time series were then Gaussian filtered using ImageJ Fiji with a standard deviation (sigma factor) of 7 pixels. Heterogeneity maps were generated using custom MATLAB code. Briefly, local mean maps were generated using the *imfilter* function and a normalising circular kernel. Intensity values were then subtracted from the mean maps. The differences maps were then divided by the local means map image. MATLAB code is available at Malvick17/HeterogeneityMaps (github.com) and on MATLAB File Exchange. For the Gaussian filter, we chose a standard deviation (sigma) of 7 pixels diameter (∼13.7 µm in myosin data, ∼8.9 µm in podosomes data) based on the area in which noncontiguous waves were observed. For the heterogeneity filter, we chose a kernel of 8 pixels (∼1.7 µm) based on the size of the NMII puncta and 16 pixels, 2.24 µm in diameter to consider the size of F‐actin podosomes.

### 4.6 STICS analysis

STICS analysis was performed using the latest version of the ICS Tools package, which we plan to publish and make freely available soon. STICS parameters are listed below:

**Table:** 

	Myosin			Podosomes/simulations		
Parameter	Original	Gaussian filtered	Heterogeneity maps	Original	Gaussian filtered	Heterogeneity maps
ROI size (px)	32	32	16	32	32	32
ROI shift (px)	4	4	2	4	4	4
TOI size (frames)	5	5	5	5	5	5
TOI shift (frames)	1	1	1	1	1	1
Kernel size	N/A	N/A	8	N/A	N/A	16
Gaussian SD	N/A	7	N/A	N/A	7	N/A

## AUTHOR CONTRIBUTIONS

HUVEC image acquisition: RMR and AH. Data analysis: RMR. Figure preparation: RMR with input from all other authors. Project supervision: AH, PWW and KD. Discussion and interpretation of the results: RMR, KD, AC, PWW and AH. RMR and KD wrote the manuscript with input from all authors.

## CONFLICT OF INTEREST STATEMENT

The authors declare no conflicts of interest.

## Supporting information

Supporting information

Supporting information

Supporting information

Supporting information

Supporting information

Supporting information

Supporting information

Supporting information

Supporting information

Supporting information

Supporting information

Supporting information

Supporting information

Supporting information

Supporting information

Supporting information

Supporting information
